# Editorial: Genetic and epigenetic regulation of stem cells by the immune system in homeostasis, regeneration, and oncogenesis

**DOI:** 10.3389/fgene.2023.1230881

**Published:** 2023-06-14

**Authors:** John M. Perry, Pengxu Qian

**Affiliations:** ^1^ Children’s Mercy Kansas City, Kansas City, MO, United States; ^2^ Department of Pediatrics, University of Kansas Medical Center, Kansas City, KS, United States; ^3^ Department of Pediatrics, University of Missouri Kansas City School of Medicine, Kansas City, MO, United States; ^4^ Center of Stem Cell and Regenerative Medicine, Bone Marrow Transplantation Center of the First Affiliated Hospital, Zhejiang University School of Medicine, Hangzhou, China; ^5^Liangzhu Laboratory, Zhejiang University Medical Center, Hangzhou, China; ^6^ Institute of Hematology, Zhejiang University and Zhejiang Engineering Laboratory for Stem Cell and Immunotherapy, Hangzhou, China

**Keywords:** stem cells, oncoimmunology, cancer, epigenetics, immunotherapy

The long-running “war on cancer” has yielded mixed but often disappointing results. This is often due to the development of resistance to therapies, which results in relapse. Often, therapeutic resistance and relapse are a consequence of the hijacking of stem cell programs by persistent subsets of cancer cells. Programs conferring “stemness” are regulated not only by genetic but especially epigenetic mechanisms. Stemness mechanisms include long-term proliferation potential, cell survival or anti-apoptosis, drug resistance, and potentially immune privilege. Furthermore, the stem cell microenvironment plays key roles in maintaining homeostasis, regeneration in response to injury, and the corruption of this capacity by cancer stem cells. This Research Topic seeks to improve understanding of the genetic and epigenetic regulation of these processes to create novel or improved strategies for targeting therapeutic resistance and relapse.

In this Research Topic, Liu and Li reviewed the role of PTIP-associated protein 1 (PA1) in the histone methyltransferases MLL3/4. Among other critical biological processes, PA1 plays essential roles in the epigenetic regulation embryogenesis. Beyond known roles in regulating mammalian histone 3 lysine 4 (H3K4) methyltransferases, PA1 interacts with transcription factors. PA1 functions as a link between lncRNA and MLL3/4 complex and in promoting the expression of CEBPA in adipogenesis. PA1 also collaborates with PTIP in transcriptional initiation of noncoding germline transcripts including Igh-γ3 through MLL3/4-independent H3K4me in B cell development. Furthermore, PA1 binds transcription factors including SMADs, CREB, and JUN. However, its other roles remain apparent but largely unknown. Future studies will provide additional information on its role in neurological development and tumorigenesis; however, Lui and Li have provided an early draft describing the current research surrounding this important protein.

Circular RNAs (circRNAs) are endogenous, closed sequences with exhibiting tissue and cell-specific expression and are sometimes abundant and evolutionarily conserved. circRNAs sometimes act as protein or microRNA inhibitors and are implicated in several diseases, including cancer. Wang et al. examine the roles of circRNAs in pathological osteogenesis, specifically in ankylosing spondylitis (AS), a chronic autoimmune disease with an incidence of approximately 2%–5% and high morbidity. Specifically, circRNA and miRNA expression profiling on osteogenically differentiated bone marrow mesenchymal stem cells (MSCs) isolated from AS patients showed significant differential expression differences from healthy controls. In fact, over 400 circRNAs showed significant changes, mainly in those regulating cell matrix adhesion and TGF-β signaling. Overall, a specific circRNA, Hsa_circ_0070562, was found to act as a pro-osteogenic factor, which is a potential biomarker and therapeutic target for AS. However, circRNAs are a relatively recent discovery and much remains to be learned regarding how they regulate osteogenesis and, much more broadly, stem cells and the immune system.

AS is also studied in this Research Topic the context of long noncoding RNAs (lncRNAs) (Cen et al.). In particular, lncRNA-mRNA networks during MSC differentiation into adipocytes from AS patients were found to be corrupted. MSCs from AS patients had previously been found to exhibit aberrantly higher adipogenic differentiation. In searching for a possible mechanism underlying this change, the authors profiled the expression of lncRNAs and mRNAs in AS MSCs vs. healthy donor MSCs. Differentially expressed lncRNAs and mRNA candidates were identified, which were related to enhanced adipogenesis via the peroxisome proliferator-activated receptor (PPAR) signaling pathway. Notably, these findings provide potential targets against fat metaplasia and aberrant osteogenesis in AS patients.

Also in MSCs, Cai et al. provide a review of mammalian target of rapamycin (mTor) function, especially regarding its role in differentiation and the unique immune response biology of MSCs. mTor is a serine/threonine kinase with extensive roles in many basic and specialized cellular functions, including survival, proliferation, metabolism, autophagy and aging. The authors summarize recent findings regarding mTor’s role in the differentiation of MSCs into adipocytes, bone and muscle tissue. They further discuss mTor’s immunomodulatory role via MSCs. Given the extensive tools available to target the mTor pathways specifically and potently, this review will be valuable for informing future development of MSCs as living and potentially self-perpetuating immunomodulators.

Finally, the role of epigenetics in the regulation of signaling in stem cells, tumorigenesis and therapeutic resistance in lung is reviewed here by Wu et al. While much is known on this subject regarding other stem cell systems, particularly hematopoietic stem cells, it is not as well-understood in lung. Nonetheless, pathological subtypes of lung cancer are determined by the differentiation state of its cells of origin. Further, therapeutic resistance often results from hijacking normal mechanisms of tissue and DNA damage repair mechanism, particularly and ultimately through stem cells. Thus, the cooption of normal stemness mechanisms by cancer stem cells in the lung can be responsible for resistance and relapse. Notably, stem cells are largely regulated through epigenetic mechanism, and, unlike genetic mechanisms, these are more readily targeted and reversible, at least in principle. Extensive future work is needed to realize this possibility, however.

Overall, this Research Topic examines the genetic and epigenetic mechanisms governing stem cells in the context of their microenvironments in homeostasis, regeneration, and oncogenesis. Continued work in these disparate but related fields will be necessary to realize the potential of regenerative medicine and targeting pathological states driven by the corruption of stem cell mechanisms.

